# CD146‐Targeted Multimodal Image‐Guided Photoimmunotherapy of Melanoma

**DOI:** 10.1002/advs.201801237

**Published:** 2019-02-27

**Authors:** Weijun Wei, Dawei Jiang, Emily B. Ehlerding, Todd E. Barnhart, Yunan Yang, Jonathan W. Engle, Quan‐Yong Luo, Peng Huang, Weibo Cai

**Affiliations:** ^1^ Department of Nuclear Medicine Shanghai Jiao Tong University Affiliated Sixth People's Hospital 600 Yishan Road Shanghai 200233 China; ^2^ Department of Radiology University of Wisconsin–Madison Madison WI 53705 USA; ^3^ Guangdong Key Laboratory for Biomedical Measurements and Ultrasound Imaging Carson International Cancer Center Laboratory of Evolutionary Theranostics School of Biomedical Engineering Health Science Center Shenzhen University Shenzhen 518060 China; ^4^ Department of Medical Physics University of Wisconsin–Madison Madison WI 53705 USA; ^5^ University of Wisconsin Carbone Cancer Center Madison WI 53705 USA

**Keywords:** immunoPET, melanoma, photoimmunotherapy, theranostics, YY146

## Abstract

For melanoma resistant to molecularly targeted therapy and immunotherapy, new treatment strategies are urgently needed. A molecularly targeted theranostic pair may thus be of importance, where the diagnostic probe facilitates patient stratification and the therapeutic companion treats the selected cases. For this purpose, flow cytometry is used to assess the CD146 level in melanoma cells. Based on YY146, a CD146‐specific monoclonal antibody, an imaging probe ^89^Zr‐Df‐YY146 is synthesized and its diagnostic performance is evaluated by positron emission tomography (PET) imaging. Furthermore, a photoimmunotherapy (PIT) agent IR700‐YY146 is developed and the therapeutic effect of IR700‐YY146 PIT is assessed comprehensively. CD146 is highly expressed in A375 and SK‐MEL‐5 cells. ^89^Zr‐Df‐YY146 PET readily detects CD146‐positive A375 melanomas. Tumor accumulation of ^89^Zr‐Df‐YY146 peaks at 72 h with an uptake value of 26.48 ± 3.28%ID g^−1^, whereas the highest uptake of the nonspecific ^89^Zr‐Df‐IgG is 4.80 ± 1.75%ID g^−1^. More importantly, IR700‐YY146 PIT effectively inhibits the growth of A375 tumors, owing to production of reactive oxygen species, decreased glucose metabolism, and reduced expression of CD146. To conclude, ^89^Zr‐Df‐YY146 and IR700‐YY146 are a promising theranostic pair with the former revealing CD146 expression in melanoma as a PET probe and the latter specifically treating CD146‐positive melanoma as an effective PIT agent.

## 1 Introduction

Primary melanomas are heterogeneous tumors, ranging from melanocytic nevi and dysplastic nevi to malignant ones.^1^ A certain percentage of fully evolved melanomas harbor multiple pathogenic mutations, of which mutations in the MAPK signaling pathway (e.g., *BRAF* ^T1799A^) shape the genomic landscape and impact multiple cellular processes (e.g., growth, metabolism, and metastasis) of melanomas.^2^ Molecularly targeted therapy using small‐molecular inhibitors and immunotherapy using monoclonal antibodies (mAbs) or cell therapies are greatly changing the treatment landscape of melanomas. However, resistance to these novel therapies and treatment‐related adverse effects often lead to termination of the treatments and, as a result, poor outcomes in many melanoma patients.^3^

Currently, ^18^F‐fluorodeoxyglucose (^18^F‐FDG) positron emission tomography (PET) is the most commonly used imaging technique for diagnosing melanoma and for monitoring treatment response following either molecularly targeted therapy or immunotherapy.^4^, ^5^ However, ^18^F‐FDG PET has poor performance when it comes to distinguishing tumor from inflammatory tissue, and it often fails to detect sentinel lymph node metastases.^6^ To this end, various radiolabeled probes targeting molecular biomarkers or biochemical processes have been developed for detecting melanoma.^7^ Still, most of these probes provide only diagnostic capabilities with few being potentially translatable.^7^ In recent years, mAbs and antibody‐based immunoconjugates have emerged as promising candidates for cancer treatments. In this setting, substantial preclinical and clinical studies strongly indicate that noninvasive PET imaging using radiolabeled antibodies (immunoPET) has a valuable role in patient stratification and response assessment.^8^, ^9^ Therefore, developing antibody‐based diagnostic probes for melanoma is highly desirable and useful.

In recent years, photodynamic therapy (PDT), which generates cytotoxic species through the interaction of a photosensitizer with light, has emerged as a powerful therapeutic modality in the armamentarium for several kinds of surface cancers. Photofrin‐mediated PDT has been exploited for treating several types of cancers, such as esophageal carcinomas,^10^ and oral squamous cell carcinoma.^11^, ^12^ Clinically, achieving a high concentration of the photosensitizer in tumors may require intratumoral injection of the photosensitizer. Meanwhile, photoimmunotherapy (PIT) elaborately combines a near infrared (NIR) dye IRDye 700DX (IR700) with tumor‐targeting mAbs and is increasingly being tested for cancer therapies.^13^, ^14^, ^15^ Compared to traditional PDT, PIT is a novel and precise therapeutic strategy selectively killing IR700‐mAb‐bound cancer cells.^13^ An ongoing phase 1/2a clinical trial is evaluating RM‐1929 PIT (a chemical conjugate of IR700 with cetuximab) in patients with recurrent head and neck cancers (https://clinicaltrials.gov/ct2/show/NCT02422979).

CD146 (cluster of differentiation 146) is a member of the cell adhesion molecule superfamily. While most studies have focused on exploring the role of CD146 in physiopathological processes (e.g., development and angiogenesis),^16^ CD146 was originally identified as a melanoma marker, presenting on the endothelia of blood vessels in both primary and metastatic melanomas.^17^, ^18^ Since these initial reports, growing evidence has revealed that upregulation of CD146 is associated with melanoma progression and metastasis by mediating cell motility,^19^ or by increasing angiogenesis.^20^ These studies together imply CD146 as an attractive marker for designing new therapeutic approaches and for predicting the outcome of patients with melanomas. Several CD146‐targeting antibodies, including a fully humanized antibody (ABX‐MA1),^21^ a murine antibody AA98,^22^ and a rat TsCD146 mAb,^23^ have been developed for treating melanomas in preclinical models. In previous studies, we reported that CD146 was an attractive target for designing PET and dual‐modal imaging probes for several types of cancers. We initially generated and radiolabeled an anti‐CD146 mAb (YY146) for PET imaging of glioblastoma.^24^, ^25^ Noninvasive molecular imaging approaches based on YY146 could detect subcutaneous and metastatic lung cancer xenografts,^26^, ^27^ and enable dual‐modal imaging and image‐guided resection of orthotopic hepatocellular carcinoma as well.^28^

In this study, we set out to develop a pair of CD146‐specific theranostic agents for detecting and treating melanomas. First, we evaluate the expression of CD146 in melanoma cell lines. Then, we radiolabel YY146 with ^89^Zr because of its long physical half‐life (78.4 h) and use the developed probe ^89^Zr‐Df‐YY146 for PET imaging of melanomas. To develop a CD146‐targeted therapeutic companion, we further conjugate YY146 with IR700 and assess the treatment efficacy of IR700‐YY146 PIT in preclinical A375‐bearing mouse models. Our results suggest that CD146‐targeted theranostic pair may customize both diagnostic and therapeutic strategies for recalcitrant melanomas.

## 2 Results

### 2.1 CD146 is Overexpressed in Melanoma Cell Lines

**Figure 1:**
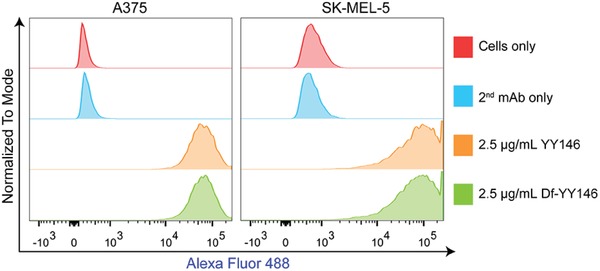
Flow cytometry histogram of A375 and SK‐MEL‐5 cells incubated with YY146, Df‐YY146, or without primary and/or secondary antibodies.

We first performed flow cytometry study to detect the cell surface expression of CD146 in two melanoma cell lines, A375 and SK‐MEL‐5. As shown in **Figure** **1**, both the two cell lines tested were CD146‐positive where YY146 was used as the primary antibody. Conjugation of Df chelators to YY146 did not impact the binding affinity of Df‐YY146 to CD146, evidenced by similar fluorescence intensity in the two groups. In contrast, no positive staining intensity was observed when the cells were incubated only with secondary antibody (2^nd^ mAb only) or without antibodies (cells only).

### 2.2 ^89^Zr‐Df‐YY146 PET Imaging and Immunofluorescence Staining Studies

**Figure 2:**
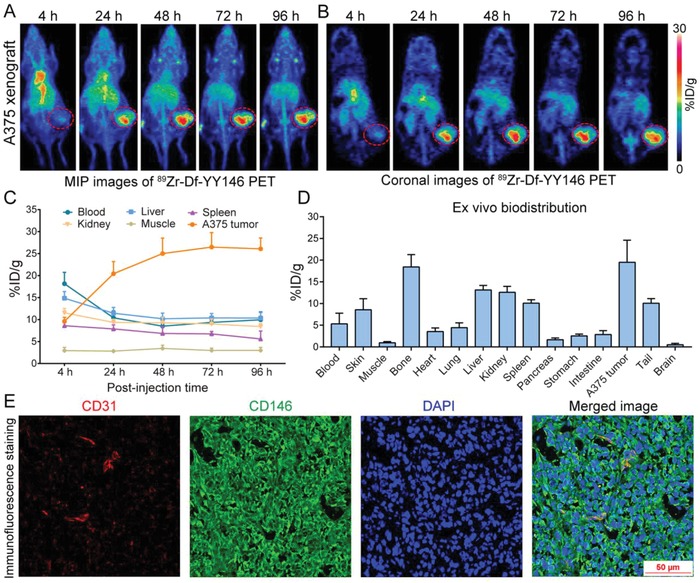
^89^Zr‐Df−YY146 PET imaging enabled clear visualization of CD146‐expressing A375 xenografts. A,B) Representative maximum intensity projection (MIP) images and coronal PET images at varying intervals post‐injection of ^89^Zr‐Df−YY146. The position of the tumor is indicated by a red dashed circle. C) Time‐activity curves showing the uptake of ^89^Zr‐Df−YY146 in the tumors and other major organs at different imaging time‐points. D) Ex vivo biodistribution data obtained at 96 h following injection of ^89^Zr‐Df−YY146. E) CD31/CD146/DAPI triple‐staining of the resected A375 tumor. Immunofluorescence staining results showed intense expression of CD146 on the surface of A375 cells accompanied by co‐expression of CD31 and CD146 on the endothelial cells. %ID g^−1^  =  percent of injected dose per gram of tissue.

^89^Zr‐Df‐YY146 was synthesized with radiochemical purity and yield above 90%. We established subcutaneous (s.c.) melanoma models using the A375 cell line, and evaluated the biodistribution profiles and tumor detection efficacy of ^89^Zr‐Df‐YY146 via PET imaging at 4, 24, 48, 72, and 96 h post‐injection (p.i.) of the radiotracer. From the maximum intensity projection (MIP) and coronal images shown in **Figure** **2**A,B, we found that ^89^Zr‐Df‐YY146 PET imaging specifically and clearly delineated A375 tumors. ^89^Zr‐Df‐YY146 also showed accumulation in the blood pool, liver, spleen, kidney, and bone joints. Uptakes in these sites were considered normal except the accumulation of unbound ^89^Zr at bilateral joints. Quantification of PET data by analyzing region‐of‐interests (ROIs) demonstrated dynamic changes of the tracer in major organs at different time‐points following injection of the probe (Figure 2C). We observed a continuous accumulation of the tracer into the tumors within the first 72 h, with a peak uptake of 26.48 ± 3.28%ID g^−1^ achieved at 72 h (*n* = 4). With the time‐dependent accumulation of ^89^Zr‐Df‐YY146 in tumors, the radioactivity in blood pool, liver, spleen and kidney gradually declined. Specifically, the liver uptakes of ^89^Zr‐Df‐YY146 at 4, 24, 48, 72, and 96 h were 14.85 ± 1.54, 11.45 ± 1.31, 10.18 ± 1.30, 10.38 ± 1.0, and 10.35 ± 1.26%ID g^−1^, respectively (*n* = 4). Because of the relatively slow clearance of full‐length antibody‐based radiotracers, the central parenchymal organs, especially the liver, may receive a high radiation dose. In clinical applications of antibody‐based PET tracers, lower administered ^89^Zr activity may result in significant reductions in radiation doses.^29^ Ex vivo biodistribution studies demonstrated an average tumor uptake of 19.52 ± 6.13%ID g^−1^ at 96 h (Figure 2D). Bone uptake was caused by the unbound or detached free ^89^Zr that preferentially accumulated in the bones.^30^ These results demonstrate that ^89^Zr‐Df‐YY146 PET is a very promising imaging technique to delineate CD146‐positive melanomas.

To confirm the specific binding of YY146 to A375 cells in vivo, tumors were harvested and tumor sections were stained for CD31, CD146, and nuclei (Figure 2E). CD31 and CD146 costaining of the tumor sections showed substantial expression of CD146 in A375 cells with abundant extracellular expression of the marker. Comparison of CD146 staining with that of CD31, a pan‐endothelial marker, showed the concomitant expression of CD146 on the endothelial cells in tumor blood vessels, in accordance with the fact that CD146 could interact with vascular endothelial growth factor receptor 2 on the endothelial cells.^31^ The immunofluorescent findings corroborated the in vivo imaging data of ^89^Zr‐Df‐YY146 PET and warranted further translational application of this tracer in melanomas.

### 2.3 ^89^Zr‐Df‐IgG PET Imaging and Biodistribution Studies

**Figure 3:**
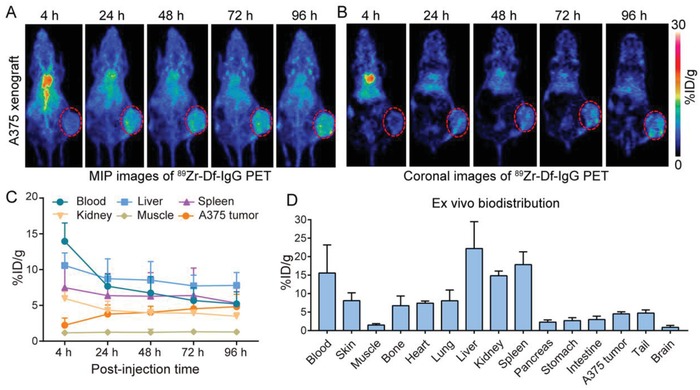
Serial ^89^Zr‐Df−IgG PET imaging of A375‐bearing nude mice. A,B) Representative maximum intensity projection (MIP) images and coronal images at varying intervals post‐injection of ^89^Zr‐Df−IgG. The tumor is indicated by a red dashed circle. C) Time‐activity curves showing uptake of ^89^Zr‐Df−IgG in the major organs and also in the tumors. D) Ex vivo biodistribution data obtained at 96 h following injection of ^89^Zr‐Df−IgG. %ID g^−1^  =  percent of injected dose per gram of tissue.

To directly compare the in vivo imaging ability of ^89^Zr‐Df‐YY146 with the nonspecific radiotracer, we did head‐to‐head comparison by investigating the imaging performance of ^89^Zr‐Df‐IgG in A375‐bearing mice (**Figure** **3**A,B). ROI analysis of the PET data is shown in Figure 3C. The tumor accumulation of ^89^Zr‐Df‐IgG peaked at 96 h with an uptake of 4.80 ± 1.75%ID g^−1^. In addition, the differences in tumor uptake between ^89^Zr‐Df‐YY146 and ^89^Zr‐Df‐IgG were statistically significant at the first and last imaging time‐points (9.60 ± 0.91 vs 2.23 ± 1.00%ID g^−1^ at 4 h, *p* < 0.0001; 26.08 ± 2.46 vs 4.8 ± 1.75%ID g^−1^ at 96 h, *p* < 0.0001; *n* = 4 for each group). In concert with the ROI data, biodistribution data obtained at 96 h p.i. of ^89^Zr‐Df‐IgG showed a tumor uptake of 4.53 ± 0.56%ID g^−1^, with a relatively higher uptake of the tracer in the liver and spleen. Collectively, these imaging results indicate that ^89^Zr‐Df‐YY146 PET, but not ^89^Zr‐Df‐IgG PET, has the ability to noninvasively detect CD146‐positive melanomas and to precisely select appropriate cases for subsequent CD146‐targeted therapies.

### 2.4 IR700‐YY146 PIT of A375 Cells

**Figure 4:**
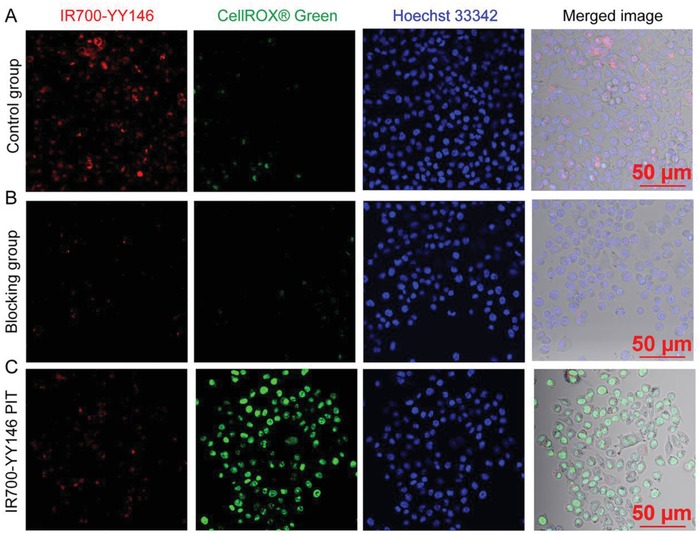
In vitro live cell imaging of A375 cells after different interventions. A) Representative staining patterns of A375 cells in the control group where the cells were incubated with 5 µg mL^−1^ of IR700‐YY146 for 6 h at 37 °C before costaining using CellROX Green and Hoechst 33342. B) Representative staining patterns of A375 cells in the blocking group where the cells were pre‐incubated with 5 µg mL^−1^ of YY146 for 2 h before IR700‐YY146 incubation and laser irradiation. C) Representative live cell imaging of A375 cells incubated with IR700‐YY146, followed by NIR irradiation. All the samples were then stained with Hoechst 33342 (5 µg mL^−1^, blue) and CellROX Green dye (5 µg mL^−1^, green) for 30 min before confocal imaging.

**Figure 5:**
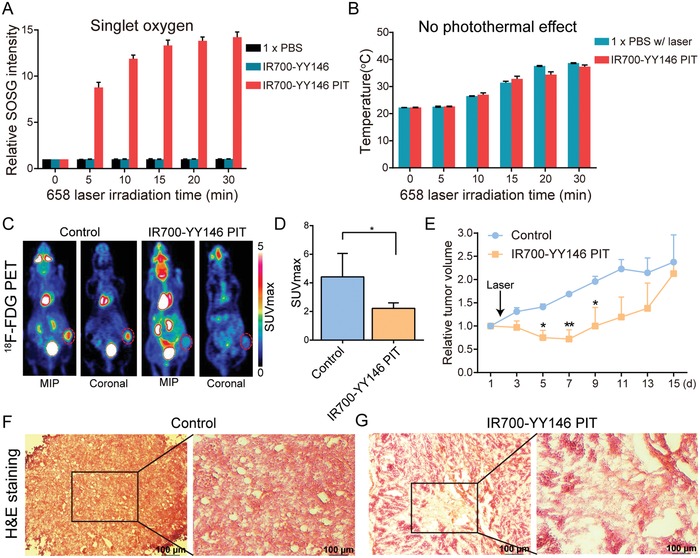
IR700‐YY146 PIT of large melanomas. A) In vitro measurement of singlet oxygen. B) In vitro measurement of photothermal effect mediated by IR700‐YY146 PIT. C) Representative ^18^F‐FDG images of the control group and IR700‐YY146 PIT group obtained 5 days after the therapy. Maximum intensity projection (MIP) and coronal images of ^18^F‐FDG PET showed different uptake patterns of the tracer in large melanomas following IR700‐YY146 PIT or saline injection. Tumors were indicated by red dashed circles. D) Quantitative analysis of tumor ^18^F‐FDG uptake in the two groups (*p < 0.05, *n* = 4 for each group). E) Tumor growth curves of nude mice bearing large A375 tumors after injection of saline or after IR700‐YY146 PIT (*p < 0.05, **p < 0.01, *n* = 4 for each group). F,G) H&E staining of the A375 tumor sections from the control group and the IR700‐YY146 PIT group. Note that H&E images were acquired at different magnifications (4 × for the regular H&E images and 10 × for the insets).

Encouraged by the above CD146‐targeted PET imaging results, we further designed a PIT probe (denoted as IR700‐YY146) and explored the therapeutic effect of this agent in vitro. Reactive oxygen species (ROS) have been implicated in the phototoxicity induced by PIT.^13^ To verify IR700‐YY146 PIT of A375 cells may trigger ROS production, we first carried out cellular tests under various conditions and used confocal imaging to monitor the effects. In consistent with previous reports,^32^, ^33^ strong red fluorescence from IR700‐YY146 was observed both on the cell surface and inside the A375 cells after incubation of IR700‐YY146 at 37 °C for 6 h, indicating extracellular binding of IR700‐YY146 and then gradual internalization of the agent into cytoplasm. Costaining with CellROX Green reagent showed that there was negligible green signal from ROS in this group without NIR irradiation (**Figure** **4**A). Pre‐incubation of A375 cells with 5 µg mL^−1^ of YY146 significantly reduced the binding and internalization of the IR700‐YY146 conjugate, thus leading to decreased accumulation of IR700‐YY146 as well as reduced ROS production in A375 cells following laser irradiation with a power density of 4 J cm^−2^ (Figure 4B). In the group where the A375 cells were first incubated with IR700‐YY146 and then exposed to laser, NIR irradiation reduced both the extracellular and intracellular red signal, and resulted in an intense increase of the green signal, indicating photobleaching of IR700‐YY146 and generation of ROS following IR700‐YY146 PIT (Figure 4C). In addition, singlet oxygen increased significantly in a time‐dependent manner in A375 cells under IR700‐YY146 PIT, whereas no such increase was detected in the phosphate buffered saline (PBS) group or in the IR700‐YY146 intervention group without laser irradiation (**Figure** **5**A). It is notable that no photothermal effect was observed in the IR700‐YY146 PIT formula (Figure 5B). These results demonstrated that IR700‐YY146 could specifically bind to and internalize into A375 cells. And IR700‐YY146 PIT could induce noticeable ROS (including singlet oxygen) production, which may potentially provide a therapeutic window of opportunity in melanomas.^34^ Hence, these results justify further assessment of IR700‐YY146 PIT in vivo in A375 models.

### 2.5 IR700‐YY146 PIT of Large Melanomas

Given the high tumor uptake of ^89^Zr‐Df‐YY146 and considerable cell internalization of IR700‐YY146, we further evaluated the therapeutic efficacy of IR700‐YY146 PIT in vivo. We first evaluated the treatment efficacy of IR700‐YY146 PIT in relatively larger A375 xenografts (average tumor volume was 330.80 ± 36.12 mm^3^, *n* = 8). We assessed the therapeutic effects by serial fluorescence imaging, tumor volume measurements, and ^18^F‐FDG PET imaging. After intravenous injection of 100 µg of IR700‐YY146 to each A375‐bearing mouse, serial IR700 fluorescence imaging was obtained. The results showed that the tumor/muscle fluorescence intensity ratio increased within the first 24 h (Figure S1, Supporting Information). The right flanks were then irradiated with NIR light (658 nm, 180 J cm^−2^) and subsequent fluorescence imaging showed a significant signal reduction in the irradiated tumor areas (Figure S1, Supporting Information). ^18^F‐FDG PET performed 5 days after the therapy showed that, when compared to the control group, the glucose metabolism as indicated by SUVmax reduced significantly in the IR700‐YY146 PIT group (4.43 ± 0.82 vs 2.23 ± 0.19, *p* < 0.05, *n* = 4 for each group; Figure 5C,D). Moreover, tumor growth was monitored by caliper measurements and the corresponding growth curves are shown in Figure 5E. For mice treated with IR700‐YY146 PIT, significant tumor reduction was observed in the first week after initiation of the therapy. However, the tumor volumes began to increase from day 9 onward and tumor volumes between the two groups had no statistical difference thereafter. Histopathological analysis of the tumors collected at day 15 showed fibrosis but also viable tumor cells in the surrounding tumor areas in the IR700‐YY146 PIT group (Figure 5F,G).

### 2.6 IR700‐YY146 PIT of Small Melanomas

**Figure 6:**
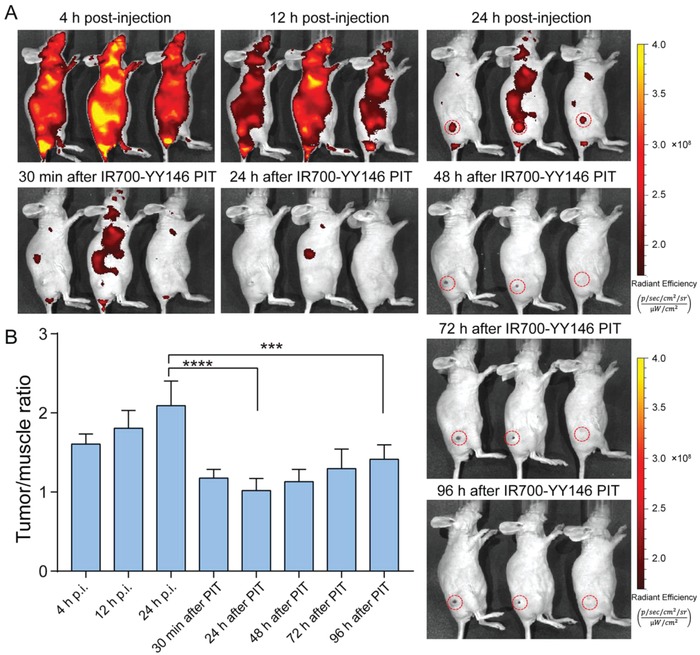
Serial IR700 fluorescence imaging before and after IR700‐YY146 PIT of small melanomas. A) Fluorescence imaging using an IVIS Spectrum was performed at different time‐points before and after NIR irradiation. Tumors were indicated by red dashed circles. B) The mean fluorescence intensity ratios of tumor to muscle were quantitatively calculated before and after IR700‐YY146 PIT (***p < 0.001, ****p < 0.0001, *p* = 4 for the group).

**Figure 7:**
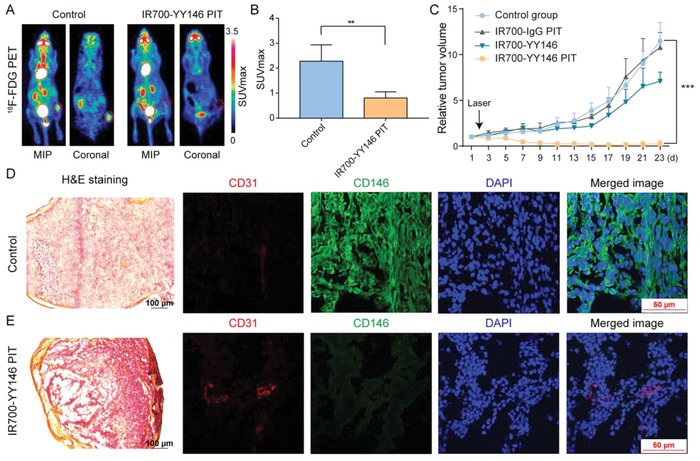
IR700‐YY146 PIT of small melanomas. A) ^18^F‐FDG PET imaging was performed 5 d after IR700‐YY146 PIT to compare glucose metabolism between the tumors in the control group and in the IR700‐YY146 PIT group. A representative tumor in the IR700‐YY146 PIT group did not show any ^18^F‐FDG uptake above background level (right panel). B) Quantitative analysis of tumor ^18^F‐FDG uptake in terms of SUVmax demonstrated statistical significance between the control group and the IR700‐YY146 PIT group. C) Tumor growth curves of A375 xenografts receiving different interventions. D) H&E and immunofluorescence staining of A375 tumors treated with saline. While H&E image showed high tumor cellularity, immunofluorescence images revealed substantial expression of CD146 in A375 cells. E) H&E and immunofluorescence staining of A375 tumors following IR700‐YY146 PIT. H&E image in this group otherwise showed extensive necrosis with scant cellularity, accompanied by reduced expression of CD146 in A375 cells revealed on immunofluorescent images. (**p < 0.01, ***p < 0.001, *n* = 4 for each group).

A previous study reported that NIR PIT induced strong decreases in volume of small brain tumors (≈50 mm^3^).^35^ Thus, we further established A375 tumors with relatively smaller volumes (86.48 ± 14.80 mm^3^, *n* = 16) and thoroughly investigated the efficacy of IR700‐YY146 PIT in these models. As shown in **Figure** **6**A, 100 µg of IR700‐YY146 was injected intravenously and tumor‐specific fluorescence signal was observed 24 h later, which was significantly reduced after NIR light exposure. Notably, obvious scabs were observed in the tumor areas 48 h after IR700‐YY146 PIT. Quantitative analysis of the fluorescence signal showed that the tumor/muscle fluorescence ratio at 24 h p.i. of IR700‐YY146 was 2.09 ± 0.31 (*n* = 4), and the corresponding ratio dropped rapidly after NIR laser exposure, indicating photobleaching in the tumors after NIR exposure (Figure 6B). Consistently, ^18^F‐FDG PET imaging was performed 5 days after the therapy to evaluate the therapeutic response (**Figure** **7**A). ^18^F‐FDG uptake in the tumors in the NIR IR700‐YY146 PIT group was lower than that of the adjacent muscle. Additional quantitative analysis demonstrated that tumor SUVmax in the IR700‐YY146 PIT group was 0.81 ± 0.11 (*n* = 4), statistically lower than that of the control group (2.28 ± 0.30, *n* = 4; *p* < 0.01; Figure 7B).

The IR700‐modified antibody conjugates, IR700‐IgG and IR700‐YY146, were homogeneous and had similar UV absorbance profiles (Figure S2, Supporting Information). A375‐bearing nude mice treated with IR700‐YY146 PIT showed significant suppression of the tumor growth, as illustrated by dynamic change of the tumor volumes in Figure 7C. After IR700‐YY146 PIT, three of the four A375 xenografts showed growth cessation and shrinkage with two of them achieved a complete response by the end of monitoring. At the end of the treatment study, IR700‐YY146 administration alone also showed therapeutic effect when compared to the control group in terms of relative tumor volume (7.08 ± 1.00 vs 11.48 ± 2.02, *p* < 0.01, *n* = 4 for each group), but this effect was less potent compared to that achieved by IR700‐YY146 PIT (7.08 ± 1.00 vs 0.24 ± 0.40, *p* < 0.0001, *n* = 4 for each group). It is not surprising IR700‐YY146 alone showed treatment efficacy, since the benefit of CD146‐targeted mAb therapy has been reported previously.^24^ Although IR700 fluorescence imaging after intravenous injection of IR700‐YY146 showed high uptake of the agent in the tumors (Figure 6A and data not shown), this phenomenon was not observed after administration of IR700‐IgG (data not shown). Consequently, IR700‐IgG PIT did not show any inhibitory effect on the growth of A375 tumors when compared to the normal control group (10.78 ± 1.66 vs 11.48 ± 2.02, *p* = 0.61, *n* = 4 for each group).

**Figure 8:**
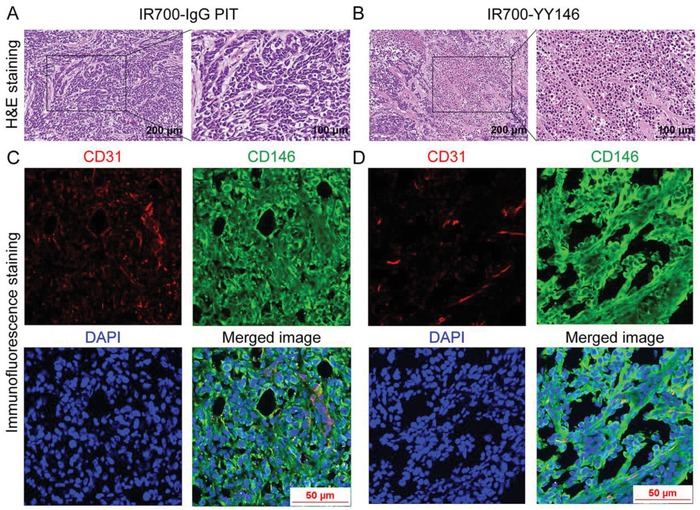
H&E and immunofluorescence staining of A375 tumors collected from IR700‐IgG PIT group and IR700‐YY146 treatment group. A) H&E image showed that the majority of the A375 tumor cells in the IR700‐IgG PIT group were viable, indicating IR700‐IgG PIT failed to exert therapeutic effect on tumor cells. B) H&E image from the IR700‐YY146 treatment group disclosed apoptotic or necrotic tumor cells inside the tumor. Immunofluorescence staining of CD31, CD146, and nuclei of tumor section from the C) IR700‐IgG PIT group and D) IR700‐YY146 treatment group. Both the two treatment options failed to reduce the expression level of CD146 in A375 cells.

While hematoxylin and eosin (H&E) staining of resected A375 tumors from the control group showed healthy and typical tumor cells with dark nuclei and eosinophilic cytoplasm (Figure 7D), the corresponding image obtained from the IR700‐YY146 PIT group revealed wide‐scale necrosis, cell debris, and fibrosis inside the tumor tissue, accompanied by lower tumor cell density (Figure 7E). Tumor sections were further stained for CD31, CD146, and nuclei. As shown in Figure 7D, immunofluorescence staining of A375 tumor sections from the control group showed intense cell surface expression of CD146. Intratumoral blood vessels delineated by CD31 staining was also observed. In comparison, immunofluorescent images showed that the level of CD146 decreased significantly in the IR700‐YY146 PIT group (Figure 7E). This observation was of importance because previous studies have reported that CD146 was an oncoprotein in melanoma.^19^ In consistent with the unsatisfactory treatment efficacy of IR700‐IgG PIT, H&E and immunofluorescence staining of the collected tumor tissue showed that the majority of the tumor cells were viable with abundant CD146 expression (**Figure** **8**A,C). While substantial dead tumor cells were noted in the IR700‐YY146‐treated tumors (Figure 8B,D and Figure S3, Supporting Information), the expression of CD146 did not alter obviously.

Taken together, these results demonstrate that CD146‐targeted NIR PIT using IR700‐YY146 may serve as a promising alternative for treating small melanomas, mainly by producing ROS and singlet oxygen, reducing glucose metabolism, and decreasing the expression level of CD146.

## 3 Discussion

Melanoma is associated with a high frequency of somatic mutations, of which a BRAF mutation in the MAPK pathway is the most common oncogenic event.^2^ However, studies have revealed that several other genetic alterations, such as mutations of CDKN2A and NRAS and loss of function of PTEN, could cooperate with mutations of the MAPK pathway (i.e., BRAF and NRAS) and synergistically give rise to melanomas of different genetic etiology.^36^ Since 2011, eight therapeutic agents, including four small‐molecular inhibitors targeting the MAPK pathway, three immune checkpoint inhibitors, and one oncolytic viral therapy, have been approved for patients with melanoma.^3^ With the approval of these agents, the treatment of metastatic melanoma has changed from a traditional palliative delay in disease progression to durable clinical responses for a large proportion of patients and a complete response for a small cohort of patients. Combination therapy consisting of a BRAF inhibitor and MEK inhibitor has become the standard‐of‐care for melanoma patients harboring the BRAF^V600E^ mutation.^3^ However, it is still difficult to achieve long‐lasting remissions for one‐third of patients, owing to the advanced stage or heterogeneity of the disease and/or drug resistance. Combination therapy using immune checkpoint inhibitors and BRAF‐targeted inhibitors is being tested in an ongoing clinical trial (NCT02224781). Even with these improvements, alternative markers that can be exploited to design theranostic approaches for melanoma are urgently needed. In such a scenario, noninvasive imaging approaches could be used to delineate melanoms in a marker‐specific manner,^7^ and marker‐targeted therapy of melanomas would become a clinical reality either as a stand‐alone therapy or in combination with clinically available therapeutics.^37^, ^38^

CD146 has been established as a pivotal biomarker in melanoma. For one, a better understanding of this marker will hopefully benefit the investigation of CD146‐related pathophysiological processes in melanomas. On the other hand, the dominant expression of CD146 in melanoma (i.e., in ≈70% of primary melanomas and 90% of lymph node metastases) renders this marker a potential candidate for identifying both primary and metastatic melanomas.^39^ Herein, we investigated and reported that ^89^Zr‐Df‐YY146 PET readily and accurately identified CD146‐positive melanomas. One can envision that this CD146‐targeted PET imaging technique could detect nodal and distant melanoma metastases since CD146‐positive circulating tumor cells have been found in late‐stage melanoma patients,^40^ and CD146 is closely associated with melanoma lung metastases.^20^ The potential clinical application of CD146‐targeted ^89^Zr‐Df‐YY146 PET is to noninvasively assess CD146 status and heterogeneity in those sites that may not be accessible by biopsy, aiding in the selection of patients for CD146‐targeted therapies in an image‐guided manner. Aside from these above‐mentioned applications, ^89^Zr‐Df‐YY146 PET imaging could also be deployed for precise delineation of resectable tumor margins.

In addition to being a diagnostic marker, CD146 is an appealing therapeutic target. To this end, we specifically investigated and reported the efficacy of IR700‐YY146 PIT in melanomas. The strong antitumor effect of IR700‐YY146 PIT herein raises the possibility that IR700‐YY146 PIT is a promising therapeutic option for early‐stage cutaneous melanomas, provided that the tumor is CD146‐positive. Moreover, we found that production of surplus ROS (including singlet oxygen), reduced glucose metabolism and suppression of CD146 expression synergistically contribute to the superior therapeutic effect of IR700‐YY146 PIT in small melanomas. Overwhelming ROS production may play a key role in mediating the treatment outcome, since Fujimoto et al. previously reported that ROS scavengers obviated the therapeutic efficacy of IR700‐mAb conjugates.^15^ Besides, production of axial ligands from IR700‐mAb conjugates and permanent plasma membrane damage are other underlying mechanisms in mediating the therapeutic effect of PIT.^41^, ^42^

Of note, our studies indicated that while we were able to achieve excellent therapeutic index for melanomas of relatively smaller volumes, the treatment efficacy for larger tumors was still not satisfactory due to the finite penetration of light into tumors. Indeed, the routine clinical application of PDT (or PIT in the coming future) lies in treating diseases located superficially such as in the skin, retina, oral cavity, and esophagus.^43^ Delivering light to large or disseminated tumors has been largely limited by attenuation in potency as the light penetrates into deep tissues, resulting in reduced efficacy of PDT in tumor tissues.^43^ However, future studies are needed to shed light on the impact of repeated IR700‐YY146 PIT and dosage of IR700‐YY146 (or laser) on the therapeutic outcome in advanced large melanomas. Furthermore, combinatorial therapies have synergistic efficacy and may overcome drug resistance encountered in single‐agent therapies. Therefore, future studies may design next‐generation therapeutics comprising of PIT and tyrosine kinase inhibitors or checkpoint inhibitors, which may lead to a synergistic reduction of tumor volume in advanced melanomas.

The PET imaging and PIT results here also lay the groundwork for other CD146‐targeted therapeutic options. For instance, developing CD146‐targeted radioimmunotherapy (RIT) for effective melanoma treatment is possible, if YY146 is radiolabeled with either beta‐emitting therapeutic radionuclide (such as ^90^Y, ^177^Lu, and ^188^Re),^44^, ^45^ or alpha‐emitting radionuclide (such as ^211^At, ^225^Ac, and ^213^Bi).^38^, ^46^ CD146‐targeted RIT will hopefully lead to tumor‐specific lethal DNA damage and enhanced immune responses.^47^ While PIT is a viable option for localized subcutaneous melanomas, radioconjugates targeting CD146 will be of great value for advanced and metastatic melanomas. Although studies have shown that Cerenkov radiation from beta emitters (such as ^64^Cu and ^18^F) can be used to excite photosensitizers (such as TiO_2_ nanoparticles) to realize deep‐tissue PDT,^48^, ^49^ future studies are needed to explore whether dual‐labeled YY146 with positron emitters (^64^Cu or ^89^Zr) and IR700 could be utilized for inducing Cerenkov radiation‐mediated PIT of melanoma.

## 4 Conclusion

We demonstrate that ^89^Zr‐Df−YY146 PET imaging is useful for delineating CD146 status in melanomas and IR700‐YY146 PIT is a promising treatment method for CD146‐positive small melanomas. Upon optimization and clinical translation, this CD146‐targeted theranostic pair may help refine clinical management of melanomas.

## 5 Experimental Section

*Cell Lines and Flow Cytometry*: A375 and SK‐MEL‐5 cell lines were kindly provided by Dr. Mark R. Albertini (University of Wisconsin‐Madison) and were cultured in phenol red free Dulbecco's Modified Eagle's Medium (DMEM, ATCC). The media were supplemented by 10% fetal bovine serum (FBS, Gibco) and 1% penicillin (100 U mL^−1^)/streptomycin (100 µg mL^−1^) solution (Invitrogen). All the cells were maintained in a humidified incubator at 37 °C and under a 5% CO_2_ atmosphere.

For flow cytometry, ≈1.0  ×  10^6^ A375 and SK‐MEL‐5 cells were harvested and washed with ice‐cold PBS (HyClone) for three times. Cells were then resuspended in flow cytometry staining buffer (Invitrogen) and stained with either YY146 (2.5 µg mL^−1^) or Df‐YY146 (2.5 µg mL^−1^) for 45 min on ice. Following the incubation of the primary antibody, cells were washed using ice‐cold PBS for three times before incubation with Alexa Fluor 488‐labeled goat antimouse IgG (5 µg mL^−1^) for 45 min. The cell samples were washed and tested using a BD LSR Fortessa flow cytometer (BD Biosciences). Flow results were analyzed with FlowJo software (FlowJo LLC).

*Subcutaneous (s.c.) A375 Melanoma Models*: All animal studies were conducted in accordance with a protocol approved by the University of Wisconsin Institutional Animal Care and Use Committee. For s.c. melanoma models, athymic female nude mice (Envigo) aged between 4–6 weeks were implanted with 2 ×  10^6^ A375 cells, which were suspended in 100**–**150 µL of a solution containing a 1:1 mixture of Matrigel (Corning) and sterile PBS. Tumor sizes were monitored every other day using a caliper. Nude mice with tumor volumes of 150**–**300 mm^3^ were used for in vivo PET imaging. For NIR PIT studies, A375‐bearing nude mice with tumor volume of ≈300 mm^3^ or ≈80 mm^3^ were used accordingly.

*Radiolabeling of YY146 and IgG with ^89^Zr*: CD146‐specific mAb YY146 was produced and purified as previously described.^24^ Detailed procedures for conjugating *p*‐SCN‐Bn‐deferoxamine (Df) to YY146 or the nonspecific human IgG1 control (Invitrogen) were reported previously.^25^ Briefly, 2 mg of mAb in PBS was added to 0.1 m Na_2_CO_3_ to reach a final pH of 8.5**–**9.0. Df was dissolved in DMSO and immediately added to the above antibody solution with a Df:YY146 molar ratio of 10:1. The final pH of the reaction media was adjusted to 8.5**–**9.0 and the reaction was incubated at room temperature for 2 h, followed by purification using PD‐10 desalting columns (GE Healthcare). Df‐YY146 was then radiolabeled using ^89^Zr, which was produced using a GE PETrace biomedical cyclotron as previously reported.^50^ ^89^Zr‐Df‐YY146 was purified and separated from free ^89^Zr using PD‐10 columns with PBS as the mobile phase.

*Small Animal PET Imaging and Data Analysis*: For PET imaging, 3.7**–**5.6 MBq of ^89^Zr‐Df‐YY146 or ^89^Zr‐Df‐IgG (about 30–50 µg of mAb) in 200 µL sterile PBS was injected via the tail vein per mouse. Before scanning, mice were anesthetized with isoflurane (2.5% for induction and 1.5% for maintenance) and placed in the prone position in the scanner. All PET images were acquired using an Inveon µPET/µCT scanner (Siemens Medical Solutions USA, Inc.) at 4 h, 24 h, 48 h, 72 h, 96 h p.i. of the tracers. For mice in the therapeutic studies, ^18^F‐FDG PET imaging was used during treatment to evaluate the glucose metabolism of the tumors according to a reported procedure with minor modifications.^51^ Briefly, mice were fasted for 12 h with access to drinking water before ^18^F‐FDG injection. Mice were kept warm on a heating pad and anesthetized using isoflurane inhalation anesthesia (2.5% in 100% oxygen). Then, ^18^F‐FDG (7.4 MBq of activity in 200 µL PBS) was injected via the tail vein, followed by a 1 h uptake period (during which the mice were kept under anesthesia to decrease muscle uptake of ^18^F‐FDG) and static PET imaging.

PET data were reconstructed using the Inveon Research Workplace (Siemens Preclinical Solutions) with a non‐scatter‐corrected 3D‐ordered subset expectation optimization/maximum a posteriori (OSEM3D/MAP) algorithm. For ^89^Zr‐Df‐YY146 and ^89^Zr‐Df‐IgG data, ROIs were delineated on the PET images and percent of injected dose per gram of tissue (%ID g^−1^) was calculated for corresponding organs and tumors. For ^18^F‐FDG PET data, tumor regions were manually drawn and tracer uptake was quantified as standardized uptake value (SUV) using the following formula: SUV = tissue activity concentration (MBq mL^−1^)/injected dose (MBq) × body weight (g).

*Ex Vivo Biodistribution Studies*: After ^89^Zr‐Df‐YY146 and ^89^Zr‐Df‐IgG PET imaging at the last time‐point, biodistribution studies were performed to quantify uptake of the tracer in relevant organs. Briefly, mice were first sacrificed by asphyxiation with CO_2_. Then, blood and chosen organs including tumors were collected, wet weights of each organ were measured, and the radioactivity of each organ was counted using a calibrated γ‐counter (Perkin Elmer Inc.). Ex vivo tissue activity was then calculated and reported as %ID g^−1^ (mean ± SD).

*Photoimmunotherapy (PIT) Studies*: IRDye 700DX NHS ester (IR700) was obtained from LI‐COR Biosciences. Conjugation of IR700‐YY146 and IR700‐IgG was performed following a previous report.^13^ In brief, YY146 or human IgG1 (1 mg, 6.8 nmol) was incubated with IR700 (66.8 µg, 34.2 nmol) in 0.1 m Na_2_HPO4 (pH of 8.5) at room temperature for 2 h. The final products were purified by using PD‐10 desalting columns and the protein concentration was measured with a NanoDrop One (Thermo Scientific). In addition, the number of IR700 per mAb was measured by absorption at 689 nm by using a Cary 60 UV‐Vis Spectrophotometer (Agilent Technologies).

For in vitro NIR PIT studies, A375 cells seeded in 6‐well plates (10 × 10^4^ cells per well) were incubated with IR700‐YY146 (5 µg mL^−1^) for 6 h with or without pre‐incubation of YY146 (5 µg mL^−1^, for 2 h prior to primary incubation). The cells were then washed with PBS and DMEM culture medium was added, followed by NIR laser irradiation with 4 J cm^−2^ at a wavelength of 658 nm using a Diode Laser System (Laserglow Technologies, Toronto, Canada). After the laser irradiation, the cells were further incubated with 5 µg mL^−1^ of Hoechst 33342 (Invitrogen) for staining nuclei and 5 µg mL^−1^ of CellROX Green dye (Invitrogen) for staining ROS in the dark for another 30 min. After washing with PBS, phenol red‐free DMEM medium was added and fluorescence images were acquired with a Nikon A1R confocal microscope. The singlet oxygen was detected using Singlet Oxygen Sensor Green reagent (Invitrogen) as per the recommendation.

For in vivo PIT of large melanomas, A375‐bearing nude mice were divided into two groups at random (*n* = 4 for each group), with one group receiving saline injection and another group receiving IR700‐YY146 PIT. For in vivo PIT of small melanomas, A375‐bearing nude mice were divided into four groups at random (*n* = 4 for each group), that is, control group, IR700‐IgG PIT group, IR700‐YY146 group, and IR700‐YY146 PIT group. For the PIT groups, 100 µg of IR700‐YY146 or IR700‐IgG was intravenously injected into each mouse. At 24 h after administration, the tumors in the PIT groups were irradiated with a 658 nm laser with 180 J cm^−2^. For the mice in the control group, an equal volume of sterile PBS but no further intervention was given. For the IR700‐YY146 treatment group, each mouse was given 100 µg of IR700‐YY146 without further laser irradiation. Tumor volumes were measured every two days after the treatment. For mice receiving IR700‐YY146 or IR700‐IgG injection, fluorescent images were obtained at different time‐points before and after laser irradiation using an IVIS Spectrum (Perkin Elmer Inc.) with a 675/30 nm excitation filter and a 720/20 nm emission filter. ROIs were drawn on the fluorescent images using the Living Image 4.5.5 (IVIS Imaging Systems) and tumor/muscle signal ratios were calculated.

*Histopathology and Immunofluorescence Studies*: After biodistribution and PIT studies, tumors were collected and embedded in optimal cutting temperature (OCT) compound (Sakura Finetek USA, Inc.) or fixed in 10% paraformaldehyde. Series of 10 µm sections were sliced at multiple tumor levels and used for immunofluorescence and H&E staining as described previously.^52^ For immunofluorescence staining, frozen tissue sections were dried at room temperature for 15 min and then fixed with cold 4% paraformaldehyde for 10 min. After rinsing with PBS and permeabilizing with 0.2% Triton X‐100 for 15 min, the sections were washed again and blocked with 5% donkey serum for 1 h at room temperature, followed by incubation of the primary antibodies (10 µg mL^−1^ of YY146, and 10 µg mL^−1^ of rat antimouse CD31 antibody) overnight at room temperature. After washing with PBS, the tumor slices were incubated with 5 µg mL^−1^ of FITC‐labeled goat antimouse IgG (SouthernBiotech) and 5 µg mL^−1^ of Cy3‐labeled donkey antirat IgG (Jackson ImmunoResearch Laboratories, Inc.) for 1 h. After washing with 0.2% Triton X‐100 for 15 min and PBS for 15 min in darkness, all sections were mounted with UltraCruz Hard‐set Mounting Medium containing 1.5 µg mL^−1^ of DAPI (Santa Cruz Biotechnology). All confocal images were acquired using a Nikon A1R confocal microscope.

*Statistical Analysis*: Quantitative data were analyzed using Prism software (Version 7.0, GraphPad Software) and presented as mean ± standard deviation. In most cases, unpaired, two‐tailed Student's *t*‐tests were used to analyze the data. When there were three or more independent groups, the one‐way analysis of variance (ANOVA) was used to calculate whether there were any statistical differences between the means of different groups. In all cases, a 95% confidence level (*p* < 0.05) was considered to represent a statistical difference in the data (*p < 0.05, ** *p* < 0.01, *** *p* < 0.001, **** *p* < 0.0001).

## Conflict of Interest

The authors declare no conflict of interest.

## Supporting information

Supplementary

## References

[1] A. H. Shain , B. C. Bastian , Nat. Rev. Cancer 2016, 16, 345. 27125352 10.1038/nrc.2016.37

[2] A. H. Shain , I. Yeh , I. Kovalyshyn , A. Sriharan , E. Talevich , A. Gagnon , R. Dummer , J. North , L. Pincus , B. Ruben , W. Rickaby , C. D'Arrigo , A. Robson , B. C. Bastian , N. Engl. J. Med. 2015, 373, 1926. 26559571 10.1056/NEJMoa1502583

[3] J. J. Luke , K. T. Flaherty , A. Ribas , G. V. Long , Nat. Rev. Clin. Oncol. 2017, 14, 463. 28374786 10.1038/nrclinonc.2017.43

[4] A. N. M. Wong , G. A. McArthur , M. S. Hofman , R. J. Hicks , Eur. J. Nucl. Med. Mol. Imaging 2017, 44, 67. 28389693 10.1007/s00259-017-3691-7

[5] P. H. Vensby , G. Schmidt , A. Kjaer , B. M. Fischer , Am. J. Nucl. Med. Mol. Imaging 2017, 7, 255. 29348980 PMC5768920

[6] K. M. Acland , C. Healy , E. Calonje , M. O'Doherty , T. Nunan , C. Page , E. Higgins , R. Russell‐Jones , J. Clin. Oncol. 2001, 19, 2674. 11352959 10.1200/JCO.2001.19.10.2674

[7] W. Wei , E. B. Ehlerding , X. Lan , Q. Luo , W. Cai , Eur. J. Nucl. Med. Mol. Imaging 2018, 45, 132. 29085965 10.1007/s00259-017-3839-5 PMC5700861

[8] K. L. Moek , D. Giesen , I. C. Kok , D. J. A. de Groot , M. Jalving , R. S. N. Fehrmann , M. N. Lub‐de Hooge , A. H. Brouwers , E. G. E. de Vries , J. Nucl. Med. 2017, 58, 83S. 28864618 10.2967/jnumed.116.186940

[9] W. Wei , D. Ni , E. B. Ehlerding , Q. Y. Luo , W. Cai , Mol. Cancer Ther. 2018, 17, 1625. 30068751 10.1158/1535-7163.MCT-18-0087 PMC6168319

[10] F. J. Civantos , B. Karakullukcu , M. Biel , C. E. Silver , A. Rinaldo , N. F. Saba , R. P. Takes , V. Vander Poorten , A. Ferlito , Adv. Ther. 2018, 35, 324. 29417455 10.1007/s12325-018-0659-3

[11] C. Hopper , A. Kubler , H. Lewis , I. B. Tan , G. Putnam , Int. J. Cancer 2004, 111, 138. 15185355 10.1002/ijc.20209

[12] P. H. Ahn , H. Quon , B. W. O'Malley , G. Weinstein , A. Chalian , K. Malloy , J. H. Atkins , T. Sollecito , M. Greenberg , S. McNulty , A. Lin , T. C. Zhu , J. C. Finlay , K. Cengel , V. Livolsi , M. Feldman , R. Mick , T. M. Busch , Oral Oncol. 2016, 55, 37. 26865261 10.1016/j.oraloncology.2016.01.013 PMC4943020

[13] M. Mitsunaga , M. Ogawa , N. Kosaka , L. T. Rosenblum , P. L. Choyke , H. Kobayashi , Nat. Med. 2011, 17, 1685. 22057348 10.1038/nm.2554 PMC3233641

[14] K. Sato , N. Sato , B. Xu , Y. Nakamura , T. Nagaya , P. L. Choyke , Y. Hasegawa , H. Kobayashi , Sci. Transl. Med. 2016, 8, 352ra110. 10.1126/scitranslmed.aaf6843 PMC7780242 27535621

[15] S. Fujimoto , N. Muguruma , K. Okamoto , T. Kurihara , Y. Sato , Y. Miyamoto , S. Kitamura , H. Miyamoto , T. Taguchi , K. Tsuneyama , T. Takayama , Theranostics 2018, 8, 2313. 29721082 10.7150/thno.22027 PMC5928892

[16] Z. Wang , X. Yan , Cancer Lett. 2013, 330, 150. 23266426 10.1016/j.canlet.2012.11.049

[17] C. Sers , G. Riethmuller , J. P. Johnson , Cancer Res. 1994, 54, 5689. 7923217

[18] J. P. Johnson , M. M. Rummel , U. Rothbacher , C. Sers , Curr. Top. Microbiol. Immunol. 1996, 213, 95. 8814997 10.1007/978-3-642-61107-0_7

[19] V. O. Melnikova , K. Balasubramanian , G. J. Villares , A. S. Dobroff , M. Zigler , H. Wang , F. Petersson , J. E. Price , A. Schroit , V. G. Prieto , M. C. Hung , M. Bar‐Eli , J. Biol. Chem. 2009, 284, 28845. 19703903 10.1074/jbc.M109.042150 PMC2781430

[20] L. Mills , C. Tellez , S. Huang , C. Baker , M. McCarty , L. Green , J. M. Gudas , X. Feng , M. Bar‐Eli , Cancer Res. 2002, 62, 5106. 12208768

[21] E. C. McGary , A. Heimberger , L. Mills , K. Weber , G. W. Thomas , M. Shtivelband , D. C. Lev , M. Bar‐Eli , Clin. Cancer. Res. 2003, 9, 6560. 14695161

[22] X. Yan , Y. Lin , D. Yang , Y. Shen , M. Yuan , Z. Zhang , P. Li , H. Xia , L. Li , D. Luo , Q. Liu , K. Mann , B. L. Bader , Blood 2003, 102, 184. 12609848 10.1182/blood-2002-04-1004

[23] M. Nollet , J. Stalin , A. Moyon , W. Traboulsi , A. Essaadi , S. Robert , N. Malissen , R. Bachelier , L. Daniel , A. Foucault‐Bertaud , C. Gaudy‐Marqueste , R. Lacroix , A. S. Leroyer , B. Guillet , N. Bardin , F. Dignat‐George , M. Blot‐Chabaud , Oncotarget 2017, 8, 112283. 29348825 10.18632/oncotarget.22736 PMC5762510

[24] Y. Yang , R. Hernandez , J. Rao , L. Yin , Y. Qu , J. Wu , C. G. England , S. A. Graves , C. M. Lewis , P. Wang , M. E. Meyerand , R. J. Nickles , X. W. Bian , W. Cai , Proc. Natl. Acad. Sci. USA 2015, 112, E6525. 26553993 10.1073/pnas.1502648112 PMC4664343

[25] R. Hernandez , H. Sun , C. G. England , H. F. Valdovinos , T. E. Barnhart , Y. Yang , W. Cai , Mol. Pharmaceutics 2016, 13, 2563. 10.1021/acs.molpharmaceut.6b00372 PMC4935599 27280694

[26] H. Sun , C. G. England , R. Hernandez , S. A. Graves , R. L. Majewski , A. Kamkaew , D. Jiang , T. E. Barnhart , Y. Yang , W. Cai , Eur. J. Nucl. Med. Mol. Imaging 2016, 43, 2169. 27342417 10.1007/s00259-016-3442-1 PMC5050101

[27] C. G. England , D. Jiang , R. Hernandez , H. Sun , H. F. Valdovinos , E. B. Ehlerding , J. W. Engle , Y. Yang , P. Huang , W. Cai , Mol. Pharmaceutics 2017, 14, 3239. 10.1021/acs.molpharmaceut.7b00216 PMC5624854 28825843

[28] R. Hernandez , H. Sun , C. G. England , H. F. Valdovinos , E. B. Ehlerding , T. E. Barnhart , Y. Yang , W. Cai , Theranostics 2016, 6, 1918. 27570560 10.7150/thno.15568 PMC4997246

[29] G. A. Ulaner , S. K. Lyashchenko , C. Riedl , S. Ruan , P. B. Zanzonico , D. Lake , K. Jhaveri , B. Zeglis , J. S. Lewis , J. A. O'Donoghue , J. Nucl. Med. 2018, 59, 900. 29146695 10.2967/jnumed.117.202010 PMC6004559

[30] E. Aluicio‐Sarduy , P. A. Ellison , T. E. Barnhart , W. Cai , R. J. Nickles , J. W. Engle , J. Labelled Compd. Radiopharm. 2018, 61, 636. 10.1002/jlcr.3607 PMC6050152 29341227

[31] T. Jiang , J. Zhuang , H. Duan , Y. Luo , Q. Zeng , K. Fan , H. Yan , D. Lu , Z. Ye , J. Hao , J. Feng , D. Yang , X. Yan , Blood 2012, 120, 2330. 22718841 10.1182/blood-2012-01-406108

[32] M. Ishida , S. Kagawa , K. Shimoyama , K. Takehara , K. Noma , S. Tanabe , Y. Shirakawa , H. Tazawa , H. Kobayashi , T. Fujiwara , Mol. Cancer Ther. 2016, 15, 402. 26832799 10.1158/1535-7163.MCT-15-0644 PMC4783182

[33] T. Nagaya , A. P. Gorka , R. R. Nani , S. Okuyama , F. Ogata , Y. Maruoka , P. L. Choyke , M. J. Schnermann , H. Kobayashi , Mol. Cancer Ther. 2018, 17, 661. 29237807 10.1158/1535-7163.MCT-17-0851 PMC5935585

[34] C. R. Reczek , N. S. Chandel , Annu. Rev. Cancer Biol. 2017, 1, 79. 10.1146/annurev-cancerbio-041916-065808 PMC11875389 40034143

[35] H. Jing , C. Weidensteiner , W. Reichardt , S. Gaedicke , X. Zhu , A. L. Grosu , H. Kobayashi , G. Niedermann , Theranostics 2016, 6, 862. 27162556 10.7150/thno.12890 PMC4860894

[36] N. Cancer Genome Atlas , Cell 2015, 161, 1681.26091043

[37] E. Revskaya , A. M. Jongco , R. S. Sellers , R. C. Howell , W. Koba , A. J. Guimaraes , J. D. Nosanchuk , A. Casadevall , E. Dadachova , Clin. Cancer Res. 2009, 15, 2373. 19293257 10.1158/1078-0432.CCR-08-2376

[38] A. Norain , E. Dadachova , Semin. Nucl. Med. 2016, 46, 250. 27067506 10.1053/j.semnuclmed.2015.12.005

[39] D. E. Dye , S. Medic , M. Ziman , D. R. Coombe , Front. Oncol. 2013, 3, 252. 24069584 10.3389/fonc.2013.00252 PMC3781348

[40] E. S. Gray , A. L. Reid , S. Bowyer , L. Calapre , K. Siew , R. Pearce , L. Cowell , M. H. Frank , M. Millward , M. Ziman , J. Invest. Dermatol. 2015, 135, 2040. 25830652 10.1038/jid.2015.127 PMC4504811

[41] K. Nakajima , H. Takakura , Y. Shimizu , M. Ogawa , Cancer Sci. 2018, 109, 2889. 29949672 10.1111/cas.13713 PMC6125438

[42] K. Sato , K. Ando , S. Okuyama , S. Moriguchi , T. Ogura , S. Totoki , H. Hanaoka , T. Nagaya , R. Kokawa , H. Takakura , M. Nishimura , Y. Hasegawa , P. L. Choyke , M. Ogawa , H. Kobayashi , ACS Cent. Sci. 2018, 4, 1559. 30555909 10.1021/acscentsci.8b00565 PMC6276043

[43] S. Mallidi , S. Anbil , A. L. Bulin , G. Obaid , M. Ichikawa , T. Hasan , Theranostics 2016, 6, 2458. 27877247 10.7150/thno.16183 PMC5118607

[44] E. B. Ehlerding , C. A. Ferreira , E. Aluicio‐Sarduy , D. Jiang , H. J. Lee , C. P. Theuer , J. W. Engle , W. Cai , Mol. Pharmaceutics 2018. 10.1021/acs.molpharmaceut.8b00133 PMC6028311 29787283

[45] E. B. Ehlerding , S. Lacognata , D. Jiang , C. A. Ferreira , S. Goel , R. Hernandez , J. J. Jeffery , C. P. Theuer , W. Cai , Eur. J. Nucl. Med. Mol. Imaging 2018, 45, 123. 28821931 10.1007/s00259-017-3793-2 PMC5700843

[46] S. Poty , L. C. Francesconi , M. R. McDevitt , M. J. Morris , J. S. Lewis , J. Nucl. Med. 2018.

[47] S. Espenel , A. Vallard , C. Rancoule , M. A. Garcia , J. B. Guy , C. Chargari , E. Deutsch , N. Magne , Crit. Rev. Oncol./Hematol. 2017, 110, 13. 10.1016/j.critrevonc.2016.12.003 28109401

[48] N. Kotagiri , G. P. Sudlow , W. J. Akers , S. Achilefu , Nat. Nanotechnol. 2015, 10, 370. 25751304 10.1038/nnano.2015.17 PMC4393353

[49] N. Kotagiri , M. L. Cooper , M. Rettig , C. Egbulefu , J. Prior , G. Cui , P. Karmakar , M. Zhou , X. Yang , G. Sudlow , L. Marsala , C. Chanswangphuwana , L. Lu , L. Habimana‐Griffin , M. Shokeen , X. Xu , K. Weilbaecher , M. Tomasson , G. Lanza , J. F. DiPersio , S. Achilefu , Nat. Commun. 2018, 9, 275. 29348537 10.1038/s41467-017-02758-9 PMC5773683

[50] H. Hong , G. W. Severin , Y. Yang , J. W. Engle , Y. Zhang , T. E. Barnhart , G. Liu , B. R. Leigh , R. J. Nickles , W. Cai , Eur. J. Nucl. Med. Mol. Imaging 2012, 39, 138. 21909753 10.1007/s00259-011-1930-x PMC3228902

[51] B. J. Fueger , J. Czernin , I. Hildebrandt , C. Tran , B. S. Halpern , D. Stout , M. E. Phelps , W. A. Weber , J. Nucl. Med. 2006, 47, 999. 16741310

[52] Y. Yang , Y. Zhang , H. Hong , G. Liu , B. R. Leigh , W. Cai , Eur. J. Nucl. Med. Mol. Imaging 2011, 38, 2066. 21814852 10.1007/s00259-011-1886-x PMC3189267

