# Supplementary material for: CD146‐Targeted Multimodal Image‐Guided Photoimmunotherapy of Melanoma
Source: Adv Sci (Weinh). 2019 Feb 27;6(9):1801237. doi: 10.1002/advs.201801237 (PMC6498137; doi:10.1002/advs.201801237)
Supplement: Supplementary file 1 — Supplementary [file ADVS-6-1801237-s001.pdf]

Copyright WILEY-VCH Verlag GmbH & Co. KGaA, 69469 Weinheim, Germany, 2019.

ADVANCED SCIENCE
Open Access

Supporting Information

for *Adv. Sci.,* DOI: 10.1002/advs.201801237

CD146-Targeted Multimodal Image-Guided Photoimmunotherapy of Melanoma

Weijun Wei, Dawei Jiang, Emily B. Ehlerding, Todd E. Barnhart, Yunan Yang, Jonathan W. Engle, Quan-Yong Luo, Peng Huang,* and Weibo Cai**

Copyright WILEY-VCH Verlag GmbH & Co. KGaA, 69469 Weinheim, Germany, 2019.

Supporting Information

CD146-targeted, Multimodal Image-guided Photoimmunotherapy of Melanoma

Weijun Wei[1,2,#], Dawei Jiang[2,3,#], Emily B. Ehlerding[4], Todd E. Barnhart[4], Yunan Yang[2], Jonathan W. Engle[4], Quan-Yong Luo[1,*], Peng Huang[3,*], Weibo Cai[2,4,5,*]

[1] Department of Nuclear Medicine, Shanghai Jiao Tong University Affiliated Sixth People's Hospital, 600 Yishan Road, Shanghai 200233, China

[2] Department of Radiology, University of Wisconsin - Madison, Madison, Wisconsin 53705, United States

[3] Guangdong Key Laboratory for Biomedical Measurements and Ultrasound Imaging, Carson International Cancer Center, Laboratory of Evolutionary Theranostics, School of Biomedical Engineering, Health Science Center, Shenzhen University, Shenzhen 518060, China

[4] Department of Medical Physics, University of Wisconsin - Madison, Madison, Wisconsin 53705, United States

[5] University of Wisconsin Carbone Cancer Center, Madison, Wisconsin, 53705, United States

[#]: Contributed equally

*: Correspondence to:

Prof. Weibo Cai

E-mail: wcai@uwhealth.org

Address: Room 7137, 1111 Highland Avenue, University of Wisconsin – Madison, Madison, WI 53705-2275, United States

Prof. Peng Huang

E-mail: peng.huang@szu.edu.cn

Address: Nanhai Ave 3688, Guangdong Key Laboratory for Biomedical Measurements and Ultrasound Imaging, Carson International Cancer Center, Laboratory of Evolutionary Theranostics, School of Biomedical Engineering, Health Science Center, Shenzhen University, Shenzhen 518060, China

Prof. Quan-Yong Luo
Email: luoqy@sjtu.edu.cn
Address: Department of Nuclear Medicine, Shanghai Jiao Tong University Affiliated Sixth People's Hospital, 600# Yishan Road, Shanghai 200233, China

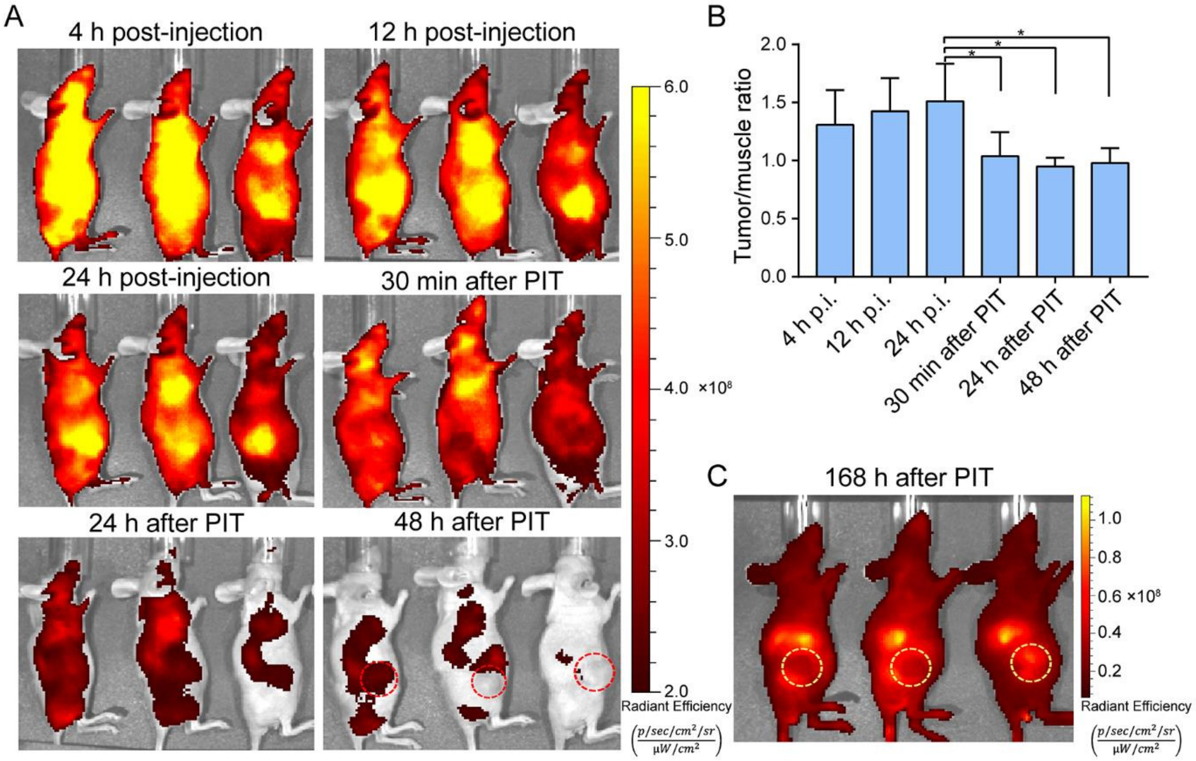

Figure S1. Serial fluorescence images of A375 melanomas with heavy tumor burden before and after NIR IR700-YY146 PIT. (A) Fluorescence images were obtained at different time-points before and after NIR irradiation. (B) The mean fluorescence ratios of tumor-to-muscle were quantitatively calculated before and after PIT (n=4 for the group, *$p < 0.05$). (C) Fluorescent images were acquired one week after NIR laser exposure. Tumors were indicated by red or yellow dashed circles.

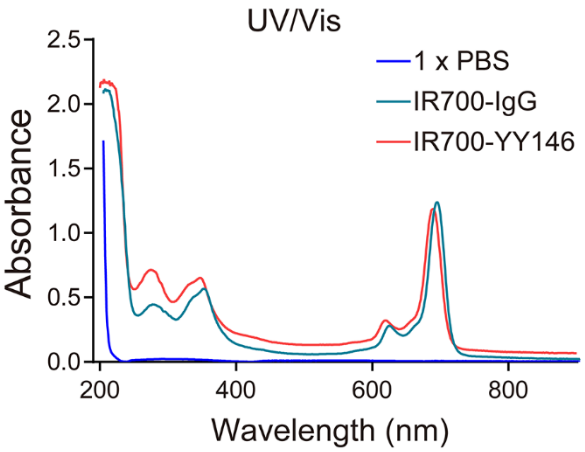

Figure S2. UV-Vis measurement of absorption profiles of the IR700-modified antibody conjugates, that is, IR700-IgG and IR700-YY146. PBS was used as a control sample.

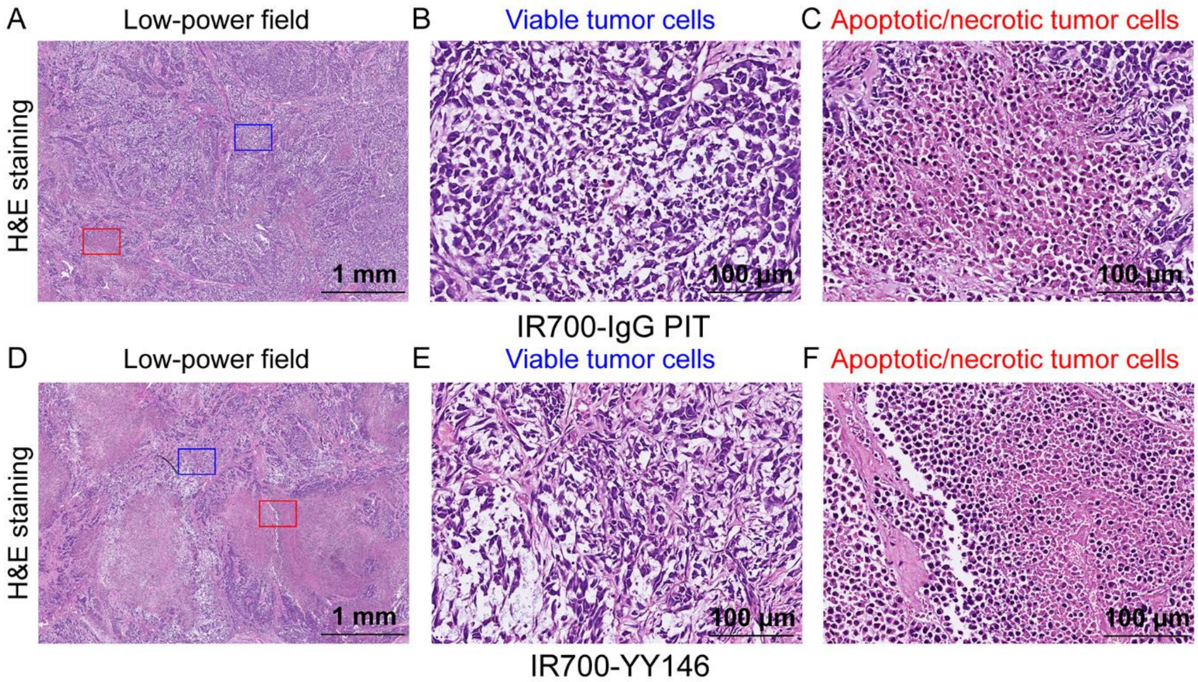

Figure S3. Hematoxylin and eosin (H&E) staining of the A375 tumors resected from mice receiving either IR700-IgG PIT or IR700-YY146 treatment. (A-C) The majority of the tumor cells were viable in the tumor specimen from the mice received IR700-IgG PIT (blue rectangle, B), while a few scattered clusters of apoptotic/necrotic tumor cells (red rectangle, C) were observed. (D-F) In comparison, H&E staining of the tumor specimen obtained from the mice receiving IR700-YY146 treatment showed large areas of cellular apoptosis or necrosis (F), which was indicative of the therapeutic effect of YY146.
